# Hendrickson Class II Palatal Fracture Following a Road Trauma Accident in a Pediatric Patient

**DOI:** 10.1155/crpe/7045357

**Published:** 2024-12-11

**Authors:** Zilefac Brian Ngokwe, Ntep Ntep David Bienvenue, Nokam Kamdem Stephane, Noubissie Audrey Sandra, Bola Antoine Siafa

**Affiliations:** ^1^Department of Oral Surgery, Maxillofacial Surgery and Periodontology, Faculty of Medicine and Biomedical Sciences, University of Yaoundé I, Yaoundé, Cameroon; ^2^Post Graduate School for Life, Health and Environmental Sciences, University of Yaoundé I, Yaoundé, Cameroon; ^3^Odontostomatology and Maxillofacial Surgery Unit, Yaoundé University Teaching Hospital, Yaoundé, Cameroon

**Keywords:** palatal fracture, pedestrian struck accidents, pediatric

## Abstract

Pediatric palatal fractures are rare clinical presentations owing to the relative plasticity of their bones. We present a case of a 3-year-old pedestrian struck male patient presenting with a mid-sagittal palatal fracture which corresponds to a Hendrickson class II fracture. Diagnosis and treatment of these rare cases are very critical to ensuring proper manducatory functions and normal facial growth in these children.

## 1. Introduction

Pediatric palatal fractures are very rare, representing 2% of all pediatric facial fractures [1].

A classification system based on the location and anatomical features of the injury has been devised by Hoppe et al. [2, 3]. The categories are shown in Table 1 below with sagittal fractures (type II) represent 7% of these fractures [3].

The fusion of the mid palatal suture occurs in people aged 15–19 years old [2, 4] with the greatest degree of sutural obliteration occurring in the third decade of life, demonstrating that the patient's chronological age is an unreliable and poor predictor of the degree of fusion [5].

In terms of etiology, pedestrian struck injuries and motor vehicle accidents were the most incriminating factors with these patients generally having a history of high velocity impacts [1, 2].

Since there is little research on the presentation and management of these fractures in the pediatric population, there are currently no classification schemes designed specifically for pediatric palatal fractures.

To the best of our knowledge, this is the youngest case of a sagittal palatal fracture reported.

## 2. Case Presentation

The pediatric unit of the Yaoundé University Teaching Hospital asked for our advice for a young male patient aged 3 years with multiple dermabrasions including facial dermabrasions following a road trauma accident. Indeed, the patient was playing and got struck down by a car before being rushed to the hospital.

**Figure 1:**
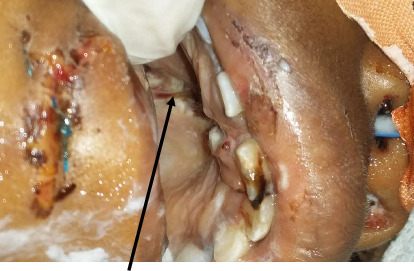
Preoperative view showing palatal laceration.

**Figure 2:**
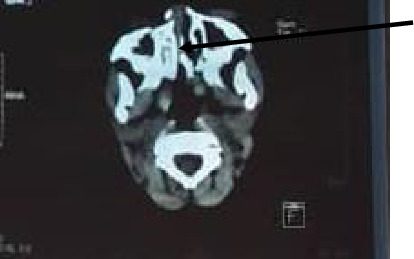
Axial view of CT scan showing sagittal fracture.

Upon examination, we observed an abnormal palatal mobility with a visible midline laceration (Figures 1 and 2) which was a palatal fracture class II according to Hendrickson (sagittal fracture) and was associated with right zygomatic fracture, a right lamina papyracea fracture, and right retro-orbital hematoma. We noted no cerebral lesions, and there was no evidence of a lesion to the cervical spine. The patient had no history of trauma, and the rest of the physical examination was without particularity.

Additionally, the patient had a nasogastric tube inserted to prevent oronasal communication.

**Figure 3:**
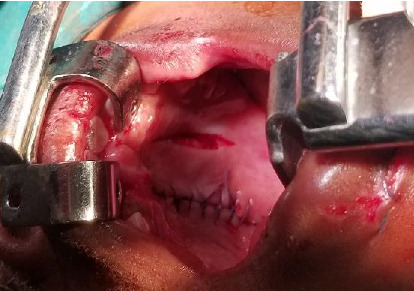
Postoperative view.

Manual surgical reduction for the palatal fracture was carried out to ensure proper alignment of the two palatine bones followed by a mucosal suturing using an absorbable 3.0 Vicryl thread as can be seen in Figure 3. No surgical plate was placed, and the clinical evolution was without event.

## 3. Discussion

Hendrickson's classification describes a documented sequence of palatal fractures, and these fractures may exist in isolation or conjunction with a broader fracture pattern. Due to the comparatively thin bone lateral to the maxilla's vomerine attachment, these fracture lines most frequently divide the palate anteroposteriorly, just off the midline [3, 6]. They pass through the maxilla's body parallel to the midline, although they typically exit between the canines in the anterior direction and either stay adjacent to it or deviate towards the tuberosity in the posterior direction [3, 6].

**Figure 4:**
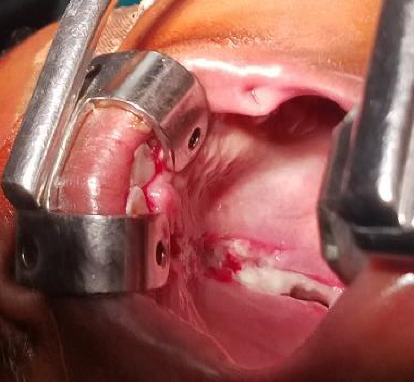
Intraoperative view.

Clinically, we observed a palatal laceration (Figures 1 and 4) which prompted us to investigate for a palatal fracture. Palatal lacerations and labial vault lacerations have been described as clinical signs associated with sagittal palatal fractures [3, 7].

This sagittal fracture is observed in children as we reported, and this could be because of the palatal suture's delayed ossification occurring during the second and third decades, which explains the tendency in younger patients [3, 8]. One more explanation for this sagittal fracture in children is the reduced midline strength in children as opposed to adults where the strength of the palatal synostosis makes this type of fractures less likely [2]. Even though skeletal plasticity in children, as well as the numerous fat pads around the jaws which cushion any impact, could result in lesser fractures perhaps accounting the far lower incidence in children [1, 9].

With respect to pedestrian struck injuries, the head, face, and neck region constituted 38% [10]. Furthermore, those who live in low- and middle-income nations, like our own, and vulnerable road users bear a disproportionate share of the burden associated with road traffic injuries [11, 12].

Sagittal fractures have also been observed to be more common in younger males [1, 8, 12] owing to their greater physical activity and susceptibility to road trauma incidents. More so 73% of all road traffic deaths occur among young males under the age of 25 years [12].

The median palatal suture serves as a growth center for the maxilla; insufficient growth at this location results malocclusion and dental crowding [13] which has an impact on manducatory functions in general.

In terms of therapeutics, given the skeletal plasticity and skeletal growth [1] as well as restricted palatal growth following repair, implications of dentition, and severe concomitant injuries upon presentation which may delay operative repair with surgical pediatric management compared to adults [1], hence, noninvasive treatment should be preferred when possible [7]. The fundamental goal of palatal fracture therapy is to restore transverse width, maintain vertical height, and anterior projection of the midface while achieving proper occlusion [8].

Surgical procedures employed include open reduction and internal fixation (ORIF) [14], transmucosal fixation using titanium miniplates [7], intermaxillary fixation, and palatal splints [1].

Transmucosal fixation is a superior choice compared to ORIF [7]. The periosteum is osteoinductive, necessary for the blood supply to the bone, and following elevation, it can form a scar. Traumatic stripping of the periosteal tissues, whether caused by direct trauma or surgical repair, has the potential to cause growth disturbances [7].

To the best of our knowledge, at 3 years of age, this is the youngest case of a sagittal palatal fracture reported.

## 4. Conclusion

Palatal fractures are rare fractures that can go unnoticed.

Pediatric patients with trauma involving the maxillofacial region should be investigated for palatal fractures given the fragility of the palatal suture.

Children are all of our future; hence, reporting of these pediatric palatal fractures will help develop a classification system and a treatment protocol to improve their overall well-being.

## Figures and Tables

**Table 1:** Hendrickson's classification of palatal fractures.

Type	Location
Type I	Alveolar
Type II	Sagittal
Type III	Parasagittal
Type IV	Para-alveolar
Type V	Complex
Type VI	Transverse

## Data Availability

Data will be available upon reasonable request from the authors.
